# Immune-inflammatory concept of the pathogenesis of chronic heart failure in dogs with dilated cardiomyopathy

**DOI:** 10.14202/vetworld.2019.1491-1498

**Published:** 2019-09

**Authors:** Yu Vatnikov, A. Rudenko, P. Rudenko, Ev Kulikov, A. Karamyan, V. Lutsay, I. Medvedev, V. Byakhova, E. Krotova, M. Molvhanova

**Affiliations:** 1 Department of Veterinary Medicine, Peoples’ Friendship University of Russia (RUDN University), Moscow 117198, Russia; 2 Department of Veterinary Medicine, Moscow State University of Food Production, Moscow 125080, Russia; 3 Laboratory of Biological Experiments, Branch of the Shemyakin-Ovchinnikov Institute of Bioorganic Chemistry of the Russian Academy of Sciences, Pushchino 117997, Russia; 4 Department of Adaptive Physical Culture and Recreation, Russian State Social University, Moscow 129226, Russia

**Keywords:** dilated cardiomyopathy, dogs, pathogenesis, heart failure, immunity and inflammation

## Abstract

**Background:**

Dilated cardiomyopathy is common in dogs. This form of cardiomyopathy is the main cause of death due to heart disease in dogs. Death can occur suddenly in clinically normal animals as a result of the progression of congestive heart failure (CHF). The pathogenesis of heart failure syndrome in dogs with dilated cardiomyopathy involves activation of the neurohumoral system and immune-mediated inflammation, which leads to further progression of the condition. Heart failure syndrome in dogs with dilated cardiomyopathy is caused by the progressive loss of cardiomyocytes, apoptosis, remodeling of the left ventricle, systolic and diastolic dysfunction, arrhythmias, reduced cerebral blood flow, the involvement of other key internal organs, and intestinal dysbiosis.

**Aim:**

This study aimed to determine the immunological and inflammatory mechanisms surrounding the development of heart failure syndrome in dogs with dilated cardiomyopathy.

**Materials and Methods:**

The subjects of this study were dogs with a dilated form of cardiomyopathy (n=159), complicated by various functional classes of heart failure syndrome. Evaluation of myocardial remodeling, systolic function, and systemic hemodynamics was performed using EMP-860 Vet and PU-2200V ultrasound scanners according to the standard technique. Electrocardiography was performed with all dogs in right lateral recumbency using the EK1T-04 Midas electrocardiograph (50 mm/s speed and 1 mV gain = 1 cm).

**Results:**

In some affected animals, especially in cases of compensated dilated cardiomyopathy, leukocytosis was noted. In patients with dilated cardiomyopathy complicated by heart failure syndrome of various functional classes, the number of neutrophils was significantly increased, and the number of lymphocytes was decreased by 1.9-2.1 times when compared with those in clinically normal animals. In dogs with dilated cardiomyopathy, neutrophilic leukocytosis develops with a simple regenerative shift to the left. The results of immunological studies indicate that dogs with dilated cardiomyopathy develop T lymphocytopenia as compared with clinically normal animals.

**Conclusion:**

The central component of heart failure syndrome in dogs with dilated cardiomyopathy is the activation of the neurohumoral system and immune-mediated inflammation. The development of CHF in dogs with dilated cardiomyopathy is caused by the progressive loss of cardiomyocytes, apoptosis, remodeling of the left ventricle, systolic and diastolic dysfunction, arrhythmias, reduced cerebral blood flow, involvement of other key internal organs, and intestinal dysbiosis.

## Introduction

Dilated cardiomyopathy is a common disease in large breed dogs [1,2] that are characterized by progressive left-sided or bilateral heart chamber dilatation and a decrease in systolic function [3,4]. As a general rule, dilated cardiomyopathy in dogs progresses rapidly, and the prognosis for the condition is unfavorable [5-7]. However, there is considerable breed variability in the rate of progression of congestive heart failure (CHF) in affected dogs [1,7].

Despite the short-term success of drug therapy in dogs with heart failure syndrome, their long-term prognosis remains poor. This determines the relevance of studies on the pathogenesis of heart failure syndrome in dogs caused by cardiomyopathy and degenerative valvular heart disease [2,4,5,8,9].

The now-dominant neurohumoral theory, which is based on the impaired function of the sympathoadrenal and renin-angiotensin-aldosterone system, does not fully explain heart failure syndrome [10-12]. Despite the effectiveness of β-adrenergic blockers, angiotensin-converting enzyme inhibitors, calcium sensitizers, and phosphodiesterase inhibitors, the course of heart failure syndrome in dogs with dilated cardiomyopathy remains progressive, which can be explained by our inability to completely block the neurohumoral system. It is also clear that in addition to the neurohormonal system, other systems must be involved in the pathogenesis of heart failure syndrome in humans and animals.

The immunological concept of the pathogenesis of chronic heart failure is based on the elevation of certain laboratory parameters (erythrocyte sedimentation rate, neutrophil numbers, C-reactive protein, and pro-inflammatory cytokines) detected in patients with cardiac disease, which is consistent with chronic aseptic inflammation [4,11-13]. These abnormalities have been confirmed in laboratory experimental models [14]. In addition to the toxic effects of the products of aseptic inflammation, stasis and hypoxia of the stomach and intestines lead to the development of malabsorption syndrome, impaired tissue metabolism, and reduced liver detoxification. All of these then lead to the development of endogenous intoxication and secondary damage to the other internal organs, which is irreversible [7,15-17].

It should be added that the question of the key factors involved in the pathogenesis of heart failure syndrome in dogs with dilated cardiomyopathy, especially in connection with other body systems, has been little studied and does not allow for definite conclusions to be made regarding the development of this type of pathology.

This study aimed to determine the immunological and inflammatory mechanisms involved in the development and progression of heart failure syndrome in dogs with dilated cardiomyopathy.

## Materials and Methods

### Ethical approval

The present study was carried out in accordance with all applicable International, National, and Institutional guidelines concerning the care and use of animals. This study was approved by the Biomedics Commission of RUDN University.

### Animals

The subjects of this study were dogs with dilated cardiomyopathy (n=159), complicated by various functional classes of heart failure syndrome. Clinically healthy, large breed dogs were used as controls.

### Collection and processing of samples

Assessment of myocardial remodeling, systolic function, and systemic hemodynamics was performed using EMP-860 Vet and PU-2200V ultrasound scanners according to the standard technique [12]. Electrocardiography was performed in all dogs, while they were positioned in the right lateral recumbency, using an EK1T-04 Midas electrocardiograph (50 mm/s speed and 1 mV gain = 1 cm).

The number of leukocytes present in the peripheral blood was counted using a Goryaev chamber; white blood cell differential counting was performed using Romanowsky–Giemsa stain. The total protein concentration of the serum was determined by the biuret reaction, protein fractions by nephelometry, blood urea nitrogen by color reaction with diacetyl monoxime, creatinine by the Jaffe reaction, bilirubin by the Jendrassik-Grof method, calcium by the Moydin and Zack method, chloride by color reaction with Argentum nitrate, potassium and sodium by flame atomic emission spectroscopy, thymol by Huergo and Popper’s method, α-amylase activity by the method of Karavei, and alanine and aspartic aminotransferase (AST) by the method of Reitman and Frankel [18]. The albumin-globulin ratio and the De Ritis ratio (AST/alanine aminotransferase [ALT]) were calculated. A BioChem SA (High Technology Inc., North Attleborough, MA 02763, USA) semi-automatic biochemical analyzer was used to perform the analyses.

The total number of T-lymphocytes present was determined using the method of Cheredeev with sheep erythrocytes and spontaneous rosetting [19]. We studied the sensitivity of T-lymphocytes to the action of theophylline [20]. The number of theophylline-sensitive T-lymphocytes (T suppressor cells) was calculated by subtracting the number of theophylline-resistant T cells (T helper cells) from the total number of T lymphocytes. The immunoregulatory index (IRI) was calculated from the ratio of T-helper cells to T-suppressor cells. The number of B-cells was calculated from the difference between the total number of lymphocytes and the number of T-lymphocytes using the method of erythrocyte rosetting [21]. The total volume of circulating immune complexes (CIC) and their fractional components was determined by molecular weight [22].

To identify the processes of autoimmunization in the test subjects, valve and myocardial autoantigens were prepared. For this purpose, tissue from the atrioventricular valves and myocardium of a clinically healthy dog with dilated cardiomyopathy that suddenly died were used. Mitral, tricuspid, and myocardial tissues were freed from the surrounding connective tissue and homogenized. Tissue autoantigens were obtained using ten freeze-thaw cycles and subsequent water-salt extraction (1:4). The suspension of crushed valvular and myocardial tissue was centrifuged for 30 min at 10,000 rpm, and the supernatant was preserved using a solution of sodium thiolate in a final concentration of 1:10,000. The resulting autoantigens were hyperimmunized in rabbits to obtain positive sera. The complement and antigens were then titrated. Autoantigens were controlled for complementarity. The complement fixation reaction was calculated using a total volume of 2.5 ml according to generally accepted methods and including sheep erythrocytes (2.5%), hemolysin (1:4), and complement, test sera, and antigens (1:3).

The amount of interleukin-1α, interleukin-6, interleukin-8, and tumor necrosis factor present in the serum of sick animals was determined using the solid-phase immunoassay method of double antibodies with sets of monoclonal antibodies and reagents (Tsitokin LLC, St. Petersburg, Russia).

### Statistical analysis

All statistical calculations were performed on a personal computer using the program STATISTICA 7.0 (StatSoft, Tulsa, Oklahoma, USA) and the following tests: Shapiro–Wilk, Kruskal–Wallis, Mann–Whitney, and Spearman’s rank correlation coefficient [23].

## Results

During the routine examination of dogs with dilated cardiomyopathy, we often identified signs of aseptic inflammation (Table-1). In some diseased animals, leukocytosis was noted, especially in cases with compensated dilated cardiomyopathy (functional Class I-II, and chronic heart failure syndrome). In dogs with dilated cardiomyopathy complicated by chronic heart failure syndrome of various functional classes, the number of band neutrophils was significantly increased (3.8-6.0 times), and the number of lymphocytes was decreased 1.9-2.1 fold as compared to those in clinically healthy animals. That is, in dogs with dilated cardiomyopathy, neutrophilic leukocytosis was seen with a simple regenerative shift to the left.

**Table 1:** Immunologic parameters of dogs with dilated cardiomyopathy according to their functional class of heart failure.

Indicator	Clinically healthy dogs, n=7	Sick dogs stratified by a functional class of chronic heart failure			

		I, n=8	II, n=8	III, n=7	IV, n=6
Leukocytes, g/l	8.9±0.65	15.7±3.36*	11.9±0.97*	13.0±1.32	11.5±1.82
Band neutrophils, g/l	0.4±0.08	2.4±0.92*	1.5±0.58	1.8±0.45*	1.5±0.21*
Segmented neutrophils, g/l	5.4±0.95	10.0±1.95	7.0±0.87	7.9±1.12	6.4±0.84
Lymphocytes, g/l	2.7±0.35	2.3±0.49	2.0±0.56	1.4±0.24*	1.3±0.19*
T-lymphocytes, g/l	0.9±0.13	1.1±0.51	0.7±0.13	0.5±0.11*	0.3±0.12*
B-lymphocytes, g/l	0.5±0.09	0.3±0.08	0.3±0.07	0.2±0.07	0.2±0.07*
O-cells, g/l	1.3±0.26	0.9±0.22	1.0±0.17	0.7±0.18	0.8±0.33
T-helper cells, g/l	0.6±0.09	0.4±0.11	0.4±0.06	0.2±0.04*	0.2±0.07*
T-suppressor cells, g/l	0.3±0.04	0.2±0.06	0.3±0.05	0.2±0.05	0.2±0.06
IRI, units	1.9±0.09	1.6±0.09	1.5±0.12*	1.4±0.09*	1.3±0.06*
CIC common, units	36.1±4.69	38.5±5.14	39.5±3.98	46.4±3.80	56.5±6.02*
CIC large, units	13.3±2.00	15.4±1.97	13.9±1.65	16.1±1.63	16.2±2.71
CIC average, units	10.6±1.34	10.6±1.50	13.4±1.65	13.4±1.65	18.0±2.39*
CIC small, units	12.3±1.92	12.5±2.01	12.3±2.01	16.9±2.69	22.0±3.02*
Interleukin-1α pg/cm^3^	1.0±0.09	1.4±0.23	1.7±0.29*	2.4±0.3*	5.4±0.7*
Interleukin-6, pg/cm^3^	1.9±1.11	5.4±2.55	14.4±1.27*	17.8±1.70*	30.3±6.05*
Interleukin-8, pg/cm^3^	1.3±0.23	3.0±0.48*	4.0±0.71	6.6±0.96*	21.0±1.86*
Tumor necrosis factor-α pg/cm^3^	2.0±1.21	1.8±0.23	3.5±0.50	9.7±2.64*	29.1±8.98*
Antifungal autoantibodies, log_2_	0.3±0.33	0.7±0.47	1.3±0.61	1.9±0.71	3.2±1.03*

* Significant difference in comparison with clinically healthy animals (hereinafter in Tables-2 and 3). CIC=Circulating immune complexes

Immunological studies have indicated that dogs with dilated cardiomyopathy develop T lymphocytopenia as compared with clinically healthy animals. A significant decrease in the absolute number of T lymphocytes in the blood of dogs with dilated cardiomyopathy, complicated by various functional classes of heart failure was identified (r=0.63; p<0.001). At the same time, analysis of variance did not reveal a significant difference in the number of B-lymphocytes between various research groups (H=5.89, p=0.21). However, the absolute number of B-lymphocytes in the blood of dogs with Class IV chronic heart failure was significantly decreased (by 2.25 times) compared with that in clinically healthy dogs. The absolute number of T-helper cells in the blood of dogs with dilated cardiomyopathy was reliably decreased (H=11.93; r=−0.54; p<0.05).

However, compared with clinically healthy dogs, the absolute number of B-lymphocytes in the blood of sick dogs was significantly lowered by 2.25 (p<0.05) times with the development of the IV functional class of chronic heart failure syndrome.

When determining the absolute number of T-helper cells in the blood of dogs with dilated cardiomyopathy, a reliable tendency was found to decrease them (H=11.93; r=−0.54; p<0.05).

The development of dilated cardiomyopathy in dogs is accompanied by significant changes to the humoral component of the immune system. In the serum of sick dogs, we found a tendency toward an increase in the level of CIC, which occurred simultaneously with an increase in the functional class of chronic heart failure (r=0.47; p<0.01). A more thorough study of the molecular composition of CIC revealed that its increased concentration in the blood of sick dogs was due to increased numbers of small and medium molecular weight fractions. We found that the serum of dogs with Class IV chronic heart failure possessed reliably increased (1.70 times, p<0.05) concentrations of medium-molecular weight CIC. The concentration of the indicated fraction of CIC in the blood of dogs with dilated cardiomyopathy was found to correlate positively with an increase in the functional class of chronic heart failure (r=0.48; p<0.01). Similarly, we found an increase in the amount of small-molecular weight CIC in the blood of sick animals. With Class IV chronic heart failure, this fraction increased significantly, from 12.3±1.92 to 22.0±3.02 units (p<0.05). The concentration of small molecule weight CIC was found to be significantly correlated with the functional class of chronic heart failure (r=0.45; p<0.01).

We established that the level of anti-myocardial complementary binding autoantibodies presents in the serum of sick dogs differed from that in clinically healthy animals. The Kruskal–Wallis test showed a tendency (H=8.62, p=0.07) toward an increase in the myocardial autoantibody titers in the blood of sick dogs with progressive chronic heart failure. In the serum of clinically healthy dogs, the level of anti-myocardial antibodies measured was 0.33 log_2_ on average. It should be added that a diagnostic titer of autoantibodies (1:5) was found in only one (14.3%) clinically healthy dog.

With the progression of disease to functional Class IV, the concentration of myocardial autoantibodies was found to be significantly (p<0.05; 9.72 times) higher than that of clinically healthy animals. At the same time, among test subjects with Class I heart failure, a diagnostic titer (1:10) of myocardial autoantibodies was detected in only one dog (12.5%). Among test subjects with Class II heart failure, a titer of 1:10 was identified in three dogs (37.5%). Among those with Class III heart failure, titers of 1:10-1:20 were found in three dogs (42.9%). Finally, among test subjects with Class IV heart failure, titers of 1:20-1:40 were identified in four dogs (66.7%). In summary, the concentration of anti-myocardial autoantibodies present in the serum of dogs with dilated cardiomyopathy was reliably correlated with the functional class of chronic heart failure (r=0.50; p<0.01).

A diagnostic titer (1:5) of autoantibodies to valvular tissue was found in the serum of only one dog with dilated cardiomyopathy (with Class IV chronic heart failure). However, in the serum of clinically healthy dogs, autoantibodies to the valvular tissue were not detected in significant amounts. Thus, the production of anti-valvular autoantibodies was not found to be a characteristic of dogs with dilated cardiomyopathy.

An increase in the activity of proinflammatory cytokines also indicates the development of an inflammatory process in the body of sick animals. In comparison with the control group, the concentrations of interleukin-1α, interleukin-6, interleukin-8, and tumor necrosis factor were significantly higher in the serum of dogs with dilated cardiomyopathy complicated by Class II-IV heart failure. We found the cytokine concentrations in dogs with dilated cardiomyopathy to be significantly correlated with the functional class of heart failure syndrome (r<0.7; p<0.001).

It has been established that the geometry of the left ventricle of dogs with dilated cardiomyopathy has been altered as compared to clinically healthy animals. In some cases, the disease manifests itself as sudden arrhythmic death syndrome. In other cases, marked chamber remodeling was noted. Our results indicate that the characteristic features of myocardial remodeling in dogs with dilated cardiomyopathy are significant dilatation and alterations in the geometry of the left ventricle, with the transition to a hemodynamically unfavorable spherical shape in addition to eccentric hypertrophy.

Left ventricular myocardial remodeling, in turn, leads to systolic and diastolic dysfunction. Thus, the ejection fraction and the degree of anteroposterior shortening of the left ventricle in sick dogs differed significantly from those of clinically healthy dogs. In clinically healthy dogs, the values of the ejection fraction and the shortening fraction averaged 60.7±1.35% (50.2-70.4) and 32.0±1.00% (0.23-0.40), respectively. In dogs with dilated cardiomyopathy complicated by Classes III and IV chronic heart failure syndrome, these values were decreased by a significant amount, 1.3-1.4 times (p<0.001) and 2.0-2.2 times (p<0.001), respectively.

In dogs with dilated cardiomyopathy, sinus tachycardia, atrial fibrillation, ventricular extrasystole, and ventricular tachycardia often occur. Sinus tachycardia was 6.8 times more likely to occur (χ^2^=5.44; p<0.05) in dogs with dilated cardiomyopathy complicated by Class I chronic heart failure than in clinically healthy animals. The likelihood of atrial fibrillation increases with the progression of heart failure syndrome in dogs with dilated cardiomyopathy. Atrial fibrillation was manifested in one (1.5%) dog with Class I, seven (23.3%) with Class II, eight (28.6%) with Class III, and 12 (54.6%) with Class IV chronic heart failure.

Regarding the development of ventricular extrasystole in dogs with dilated cardiomyopathy, it was diagnosed in five (7.6%) dogs with Class I, seven (23.3%) with Class II, three (10.7%) with Class III, and two (9.1%) with Class IV chronic heart failure.

Sinoatrial and atrioventricular block, supraventricular extrasystole, and supraventricular paroxysmal tachycardia in dogs with dilated cardiomyopathy occur sporadically. Isolated cases of such severe cardiac arrhythmias as idioventricular rhythm, flutter, and ventricular fibrillation have also been observed. These arrhythmias were mainly identified in the terminal stages of the disease during resuscitation. All dogs with these cardiac rhythm disturbances have a fatal outcome.

Left ventricular remodeling, systolic and diastolic dysfunction, and heart rhythm disturbances all lead to significant hemodynamic disturbances (Table-2). In clinically healthy dogs, left ventricular end-diastolic volume ranged from 37.8 to 102.4 cm^3^ (63.40±5.61 cm^3^). In dogs with dilated cardiomyopathy complicated by Class I-IV chronic heart failure, as compared to clinically healthy dogs, this value was increased by 1.26 (p<0.05), 1.31 (p<0.01), 1.78 (p<0.001), and 2.50 (p<0.001) times, respectively. It is also necessary to emphasize that the end-diastolic volume of the left ventricle was significantly correlated with the class of heart failure syndrome (r=0.69; p<0.001).

**Table 2:** Echocardiographic parameters of dogs with dilated cardiomyopathy, according to their functional class of heart failure, M±m, n=14-20.

Indicator	Clinically healthy dogs, n=7	Sick dogs stratified by a functional class of chronic heart failure			

		I, n=17	II, n=14	III, n=20	IV, n=16
End-diastolic volume, cm^3^	63.4±4.61	79.9±4.14*	82.8±4.07*	112.6±8.72*	158.5±12.43*
End-systolic volume, cm^3^	24.6±1.69	33.0±2.64*	36.0±3.85*	60.2±4.88*	110.9±9.03*
Stroke volume, cm^3^	38.9±3.20	46.9±3.03	46.5±1.59*	52.4±4.77*	47.5±4.88
Minute volume, cm^3^	3.9±0.37	5.3±0.49*	5.7±0.38*	7.8±0.62*	7.8±0.90*
Cardiac index, dm^3^/m^2^	3.8±0.34	4.6±0.38	5.8±0.41*	6.9±0.36*	7.3±0.80*
Ejection fraction, %	60.7±1.35	58.5±2.13	57.4±2.56	46.0±1.94*	29.7±2.13*
Mass of the left ventricle, g	123.7±5.29	116.4±5.41	118.4±5.78	130.8±6.06	163.4±6.68*

Similarly, a change in the final systolic volume of the left ventricle was noted. In the terminal stages of the disease (Classes III and IV chronic heart failure), the left-end-systolic volume increases to a greater degree than the end-diastolic volume, which indicates progressive systolic myocardial dysfunction of the left ventricle.

In dogs with dilated cardiomyopathy, the minute volume increases. Minute volume measurements in clinically healthy dogs varied ranged from 1.4 to 7.2 dm^3^, with an average of 3.9±0.37 dm^3^. In dogs with dilated cardiomyopathy complicated by Classes I-IV chronic heart failure, this value increased by 1.34 (p<0.05), 1.45 (p<0.01), 1.96 (p<0.001), and 1.97 (p<0.01) times, respectively, as compared with that in clinically healthy dogs. Interestingly, with dilated cardiomyopathy in dogs, an increase in the minute volume occurs with dilated cardiomyopathy, which is clearly associated with an increase in preload.

In dogs with dilated cardiomyopathy, the cardiac index was found to be significantly increased. In dogs with Classes II-IV chronic heart failure, this value increased by 1.52 (p<0.01), 1.82 (p<0.001), and 1.93 (p<0.01) times, respectively, as compared with that in clinically healthy dogs. The heart index in sick dogs was positively correlated with the severity of chronic heart failure (r=0.57; p<0.001). The mass of the left ventricle in dogs with severe heart failure was found to be significantly increased, indicating the development of eccentric hypertrophy.

Significant hemodynamic alterations inevitably lead to the development of multiple organ failure, as evidenced by the results of biochemical analyses performed in this study (Table-3).

**Table 3:** Biochemical parameters of dogs with dilated cardiomyopathy according to their functional class of heart failure, M±m, n=10-15.

Indicator	Clinically healthy dogs, n=7	Sick dogs stratified by a functional class of chronic heart failure			

		I, n=12	II, n=14	III, n=15	IV, n=10
Total protein, g/l	73.8±1.49	70.7±2.62	71.6±4.15	62.2±2.59*	59.3±2.95*
Albumin, g/l	36.3±0.99	35.4±1.61	35.4±1.99	29.4±0.95*	27.3±1.47*
Globulin, g/l	37.5±1.59	35.31±3.31	36.5±2.86	32.9±2.42	32.0±1.93*
Total bilirubin, μmol/l	5.1±0.54	5.6±1.17	5.1±0.68	8.6±2.41	5.5±0.61
ALT, mmol/(h×l)	0.6±0.05	0.7±0.13	1.0±0.15	1.3±0.15*	2.0±0.46*
AST, mmol/(h×l)	0.6±0.05	0.7±0.13	1.2±0.24*	1.2±0.17*	1.52±0.34*
Urea, mmol/l	6.8±0.82	10.5±2.50	8.9±0.87	11.7±2.14	19.0±3.93*
Creatinine, mmol/l	0.12±0.01	0.22±0.05*	0.16±0.02	0.17±0.03*	0.3±0.06*
α-Amylase, g/(h×l)	178.9±9.32	172.9±26.86	271.9±30.82*	183.9±29.14	452.2±72.66*
Glucose, mmol/l	4.5±0.12	4.4±0.13	5.1±0.31	4.5±0.26	5.0±0.57
Potassium, mmol/l	4.7±0.11	4.4±0.41	4.3±0.28	5.1±0.22	5.5±0.63
Calcium, mmol/l	2.6±0.17	2.2±0.21	2.5±0.06	2.2±0.05*	2.0±0.23*
Sodium, mmol/l	149.1±1.62	132.4±12.66	148.9±3.23	142.0±3.24	122.2±14.33*
Chloride, mmol/l	118.9±1.34	110.9±11.46	122.2±4.41	113.7±3.91	99.4±11.68*

ALT=Alanine aminotransferase, AST=Aspartic aminotransferase

In dogs with dilated cardiomyopathy, and especially in dogs in the final stages of heart failure, hypoproteinemia with hypoalbuminemia was noted. Failure of the liver and kidneys in sick dogs is reliably confirmed by an increase in the serum activity of alanine and aspartic transaminase, as well as an increase in the concentrations of urea and creatinine. The activity of AST was increased to a greater extent than was ALT, which can be explained by the development of cardiomyocyte cytolysis. In dogs with dilated cardiomyopathy complicated by Class IV chronic heart failure, hyponatremia, and hypochloremia were noted.

## Discussion

An important achievement over the past decade has been the development of the neurohumoral hypothesis for the progression of cardiovascular disease in humans and domestic animals [10,21,24,25]. We have established that dilated cardiomyopathy in dogs is widespread, and often familial. This form of cardiomyopathy is a leading cause of death in dogs, with death occurring suddenly in clinically healthy animals due to the progression of heart failure syndrome [2,7,26,27].

Activation of pathologic genes leads to a catastrophic decline in the energy potential of cardiomyocyte mitochondria [7,28,29]. As a result of the progressive death of cardiomyocytes, the heart weakens, and compensatory mechanisms involved in the regulation of hemodynamics are activated. These include the Frank–Starling mechanism, adrenal glands, sympathetic nervous system, renin-angiotensin-aldosterone system, antidiuretic hormone production, and the reticuloendothelial system [8,30]. An increase in the expression of aldosterone receptors in the myocardium of dogs plays an important role in the progression of dilated cardiomyopathy [31-33]. In dogs with dilated cardiomyopathy, increases in the activities of renin and aldosterone, atrial natriuretic peptide, vasopressin, cortisol, catecholamines, nitrates, and nitrites, which are the end products of nitric oxide, are seen. Activation of the renin-angiotensin-aldosterone system leads to an increase in the retention of sodium and water. This, in turn, increases preload (end-diastolic volume), which increases the total stroke volume of the heart. It is our opinion that these biologically active substances, which are synthesized by the neurohumoral system, are the main effectors in the development and progression of chronic heart failure in dogs with dilated cardiomyopathy. The constant activation of the neurohumoral system and presence of inflammation in the face of cardiomyocyte death results in myocardial remodeling, which is a structural change of the left ventricle characterized by hypertrophy, dilatation, and rounding of the heart. This process leads to a change in the geometry of the heart and further disturbances in systolic and diastolic functions [2,34,35]. Systemic inflammation plays an important role in the pathogenesis and progression of CHF. Identification of the various biomarkers of inflammation has become the subject of intense study in both human and veterinary medicine [3,24,36,37]. Histological examination often reveals inflammatory infiltrates in the myocardium of dog with dilated cardiomyopathy [12,38].

Neutrophilic leukocytosis and an increase in the serum C reactive protein levels in dogs with CHF have also been described [15,39-41]. In addition, a study conducted by Cunningham *et al*. [4] showed that both aseptic inflammation and endothelial dysfunction developed in dogs with CHF.

Studies have shown that angiotensin II increases the immunologic function of leukocytes and their adhesion to the vascular endothelium. In addition, lymphocytes and monocytes can lead to the expression of beta-adrenergic receptors, and beta-adrenergic stimulation can modulate cytokine production by these cells [42,43]. Recently, it has been established that myocardial fibroblasts in people with severe heart failure are able to synthesize pro-inflammatory cytokines [44,45]. In addition, it has been found that the expression of myocardial receptors for serotonin type 2B is associated with myocardial remodeling and cytokine synthesis in dogs with dilated cardiomyopathy [10].

We have found significant changes in the blood immunograms of dogs with dilated cardiomyopathy in our study of T-lymphocyte composition. A significant decrease in the number of T-helper cells with a relatively stable number of T-suppressor cells was identified. As a result, the incidence of ischemic-reperfusion injury (IRI) in dogs with dilated cardiomyopathy tended to decrease, which is associated with the development of an immunodeficient state. That may explain why the autoimmune processes identified in this study do not play a significant role in the pathogenesis of heart failure in dogs with dilated cardiomyopathy; they are “background” in nature and occur in response to cardiomyocyte cytolysis. It should be noted that autoantibodies developed against myocardial tissue were previously detected by immunofluorescence and Western blot analysis in the serum of dogs with dilated cardiomyopathy [1,7,45].

The processes of myocardial remodeling, arrhythmic syndrome, and systolic and diastolic dysfunction lead to impaired hemodynamics in sick dogs. The main cause of myocardial remodeling, in this case, is considered to be progressive death of cardiomyocytes due to apoptosis and the formation of replacement cardiac fibrosis [2,12,46]. In conditions of increased end-diastolic volume and left ventricular diastolic pressure, adequate forward flow is maintained using the Frank–Starling mechanism of increased contractile function. However, with significant dilatation of the left ventricle, its function deteriorates significantly, which we identified in dogs in the terminal stages of chronic heart failure (Classes III and IV).

As a result of remodeling and degeneration of myocardial tissue, areas with increased ectopic activity are created, which causes the development of various heart rhythm disorders [5,46]. According to our observations, the risk of sudden arrhythmic death syndrome in dogs with dilated cardiomyopathy increases with activation of the neurohumoral system and the development of systemic inflammation; this has been noted previously by other authors [6,39].

Blood stasis in the internal organs induces secondary damage. We identified changes in the biochemical profiles of dogs with dilated cardiomyopathy, which suggests a decrease in the protein-synthesizing function of the liver, kidney dysfunction, pancreatitis, and cytolysis of hepatocytes and cardiomyocytes. Electrolyte disorders in dogs with dilated cardiomyopathy were not pronounced.

The finding of hypoalbuminemia in dogs with dilated cardiomyopathy can be interpreted to mean that the development of CHF leads to a violation of the structure and function of the liver. That is, a hepatopathy develops, which leads to a decrease in protein biosynthesis in sick animals. In addition, a decrease in the left ventricular ejection fraction causes hemodynamic disorders and stagnation of the blood in the liver, intestines, and pancreas, which leads to indigestion and reduced flow of essential nutrients to the body of sick dogs. Clinically, this is manifested by persistent anorexia and cachexia [11,35]. The development of edema syndrome (ascites, hydrothorax, hydropericardium, and peripheral edema) significantly aggravates the hypoproteinemia due to water retention in the blood and body tissues.

Thus, in the pathogenesis of cardiomyopathies and acquired heart defects, a significant role is played by key internal organs (liver, kidney, pancreas, etc.), whose function is regularly disturbed during the development of Classes III and IV chronic heart failure. Disruption of the function of internal organs results in the development of a polymorphic pathology, that is, undoubtedly, caused by the development of chronic heart failure. Multiple organ failure was clinically more pronounced in dogs with right ventricular or biventricular heart failure.

With the development of isolated left ventricular heart failure, the function of the internal organs was impaired to a lesser extent than it was with right heart failure. Intestinal dysbiosis is also known to contribute to the activation of systemic inflammation in sick animals. This is because of the increased absorption of exotoxins and lipopolysaccharides, which are found in large quantities in bacterial cells, especially Gram-negative microorganisms.

Thus, the pathogenesis of chronic heart failure in dogs with dilated cardiomyopathy is extremely complex. This pathology results from damage and death of cardiomyocytes, myocardial remodeling, systolic and diastolic dysfunction of the left ventricle, arrhythmias, hemodynamic disorders, damage to key internal organs, and intestinal dysbiosis. It should be noted that each of these pathologic mechanisms is closely associated with the neurohumoral system and immune-mediated inflammation, and result in diverse circles of pathologic interaction.

## Conclusion

The central tenets of heart failure syndrome in dogs with dilated cardiomyopathy are activation of the neurohumoral system and immune-mediated inflammation, which further progression of the pathology. The development of CHF in dogs with dilated cardiomyopathy results from the progressive loss of cardiomyocytes, apoptosis, remodeling of the left ventricle, systolic and diastolic dysfunction, arrhythmias, reduced cerebral blood flow, the involvement of other key internal organs, and intestinal dysbiosis.

## Authors’ Contributions

YV, AR, PR, and EvK had the original idea for the study and created its design. AK, VB, EK, and MM collected the data and conducted the tests. VL, IM, AR, and PR were responsible for data analysis. YV, AK, VB, and EuK drafted the manuscript. The final draft of the manuscript was prepared by all of the authors.
